# Impact of CT Photon-Counting Virtual Monoenergetic Imaging on Visualization of Abdominal Arterial Vessels

**DOI:** 10.3390/diagnostics13050938

**Published:** 2023-03-01

**Authors:** Daniel Dillinger, Daniel Overhoff, Christian Booz, Hanns L. Kaatsch, Joel Piechotka, Achim Hagen, Matthias F. Froelich, Thomas J. Vogl, Stephan Waldeck

**Affiliations:** 1 Department of Vascular Surgery and Endovascular Surgery, Bundeswehr Central Hospital, Rübenacher Straße 170, 56072 Koblenz, Germany; 2 Department of Radiology and Neuroradiology, Bundeswehr Central Hospital, Rübenacher Straße 170, 56072 Koblenz, Germany; 3 Department of Radiology and Nuclear Medicine, University Medical Centre Mannheim, Medical Faculty Mannheim, Heidelberg University, Theodor-Kutzer-Ufer 1-3, 68167 Mannheim, Germany; 4 Institute for Diagnostic and Interventional Radiology, Goethe-University, Theodor-Stern-Kai 7, 60590 Frankfurt am Main, Germany; 5 Department of Neuroradiology, University Medical Center Mainz, Langenbeckstraße 1, 55131 Mainz, Germany

**Keywords:** computed tomography, computed tomography angiography, virtual monoenergetic imaging, contrast-to-noise ratio, signal-to-noise ratio

## Abstract

Purpose: The novel photon-counting detector (PCD) technique acquires spectral data for virtual monoenergetic imaging (VMI) in every examination. The aim of this study was the evaluation of the impact of VMI of abdominal arterial vessels on quantitative and qualitative subjective image parameters. Methods: A total of 20 patients that underwent an arterial phase computed tomography (CT) scan of the abdomen with a novel PCD CT (Siemens NAEOTOM alpha) were analyzed regarding attenuation at different energy levels in virtual monoenergetic imaging. Contrast-to-noise ratio (CNR) and signal-to-noise ratio (SNR) were calculated and compared between the different virtual monoenergetic (VME) levels with correlation to vessel diameter. In addition, subjective image parameters (overall subjective image quality, subjective image noise and vessel contrast) were evaluated. Results: Our research showed decreasing attenuation levels with increasing energy levels in virtual monoenergetic imaging regardless of vessel diameter. CNR showed best overall results at 60 keV, and SNR at 70 keV with no significant difference to 60 keV (*p* = 0.294). Subjective image quality was rated best at 70 keV for overall image quality, vessel contrast and noise. Conclusions: Our data suggest that VMI at 60–70 keV provides the best objective and subjective image quality concerning vessel contrast irrespective of vessel size.

## 1. Introduction 

Dual-energy computed tomography (CT) detector technology has been used for reconstructions with virtual monoenergetic imaging (VMI), offering further information to clinical radiologists [1,2,3,4,5,6]. Advantages of VMI are, for example, higher lesion conspicuity in oncology imaging at lower energy levels and higher attenuation, which could help in case of insufficient intravascular enhancement (e.g., in case of a missed contrast medium bolus) [7]. 

Recently, a photon-counting imaging technique using a cadmium telluride detector became usable in a clinical context [8]. The detector directly counts the photons and converts them into electric signals proportional to the energy of the photon. By assigning them to predefined energy bins, spectral information can be acquired with every examination. In addition, electronic noise can be exterminated, which improves overall image quality [8,9,10,11]. Previous studies with dual-energy technology showed that lower virtual monoenergetic (VME) keV levels, closer to the k-edge of iodine, result in higher intravascular attenuation [12,13]. Additionally, the virtual monoenergetic energy threshold of 70 keV has proven to provide better contrast-to-noise ratio (CNR), signal-to-noise ratio (SNR), and subjective image quality compared to conventional polychromatic images [14,15]. 

Higashigaito et al. carried out research on the image quality of a clinical photon-counting CT by putting a single region of interest (ROI) in the descending aorta, the portal vein, and the inferior cava vein, and comparing it to conventional polychromatic reconstruction [8]. Their images were acquired in a portal venous phase. They showed a significant increase in CNR at 50 keV in vascular structures compared to conventional imaging. They also showed a higher CNR for vascular structures at 40, 50 and 60 keV compared to the energy-integrating detector CT, and they concluded that 50 keV might be the “best tradeoff between improvement of CNR and subjective image quality of portal–venous abdominal” photon-counting CT. 

These results suggest that reconstructing VMI of photon-counting CT scans in the arterial phase might show comparable results. Our aim was to compare VME images at different energy thresholds regarding objective and subjective image quality with a particular attention to intravascular contrast enhancement. In addition, we wanted to evaluate how vessel size affects the measured attenuation, SNR and CNR at different VME energy thresholds. 

## 2. Materials and Methods

The present study was approved by the local ethics committee in Mainz, Germany (State Chamber of Physicians, number 2022-16314) and conducted in accordance with the Declaration of Helsinki.

### 2.1. Data Acquisition

We retrospectively analyzed 20 datasets of patients which underwent a contrast enhanced (CE) CT scan on a photon-counting CT scanner (NAEOTOM Alpha, Siemens Healthineers, Forchheim, Germany) between December 2021 and January 2022 which included an arterial phase of the abdominal vessels. We analyzed the arterial contrast phase of the abdomen including the iliac vessels. Exclusion criteria were arterial dissections, stent grafts, or any devices that could affect the intravascular attenuation (e.g., coil embolizations). CT scans were performed after intravenous injection of 100 mL of contrast medium (Xenetix 350, containing 76.78 g/100 mL Iobitridol, Guerbet, Roissy, France) with a flow rate of 4 mL/s followed by a bolus of 50 mL saline solution with a flow rate of 4 mL/s. The contrast medium was administered through an antecubital 18-gauge intravenous catheter using a CT Motion XD8000 (Ulrich Medical, Ulm, Germany) power injector. The scan was triggered by an ROI which was placed in the descending aorta using the CARE Bolus software (Siemens Healthineers, Forchheim, Germany). The tube current was modulated along the *z*-axis according to patient diameter and attenuation was calculated from both topograms. The tube voltage was automatically switched between 100 and 120 kV, and the scans were performed in a cranio-caudal direction. We used a pitch of 0.80 and an increment of 0.70 mm. The data were sent to a syngo server (Siemens Healthineers, Forchheim, Germany). The spectral post processing (SPP) datasets were analyzed with a slice thickness of 1 mm in a Qr40 kernel. Radiation was measured using DLP and CTDI. We also calculated patient diameter according to O’Neil et al., as they showed that it correlates directly with BMI [16]. Effective dose was calculated with Radimetrics 3.4.0 (02.02.2022) (Bayer AG, Leverkusen, Germany) according to ICRP 103 using individual Monte Carlo simulation with phantoms adapted to patient size and mAs modulation. The scan range was adapted to the phantom for every patient. The doses were verified by using a conversion factor of 0.015 for the abdomen and 0.014 for the chest according to the American Association of Physicists in Medicine [17]. 

### 2.2. Objective Image Parameters

Using syngo software version VB60A_HF04 (Siemens Healthineers, Forchheim, Germany), the following vessels were analyzed: descending aorta, coeliac trunk, superior and inferior mesentery arteries, left and right renal arteries, left and right common iliac arteries, lineal artery, gastroduodenal artery, and common and proper hepatic arteries. Three ROIs were placed in each of the vessels and the vessel diameter was measured at the same level. For the abdominal aorta, the measurements were always placed at the level of the diaphragm, the renal arteries and the aortic bifurcation. For the smaller vessels, the measurements were placed at their origin, then further along their course with at least a few millimeters distance from the prior measurements in the non-calcified, non-atherosclerotic parts of the vessels. If doubled arteries were found, the measurements were placed in the artery with the larger diameter. 

These placements were performed at 70 keV with window settings of center = 150 and width = 1200 in accordance with Rassouli et al. and Sudarski et al. [1,14]. Additionally, we also placed ROIs in the left psoas muscle and intra-abdominal bowel air to calculate signal-to-noise ratio as well as contrast-to-noise ratio. The ROIs were made as large as possible in order to minimize the influence of fatty streaks within the muscle or other relevant attenuation changes. The attenuation of these ROIs—including the standard deviation—was measured using VMI at different virtual energy levels from 40 keV to 190 keV in steps of 10 keV. 

### 2.3. Subjective Image Parameters

Two independent radiologists (ten years’ expertise and twelve years’ expertise) reviewed the images for subjective overall image quality using a five-point scale from 1 to 5 (1 = very poor, 5 = optimal), image noise (1 = major noise, 5 = none) and diagnostic assessability of vessel contrast and vessel wall (1 = not diagnostic, 5 = best prerequisites for adequate vascular diagnostics). For detailed information, see Table 1. 

### 2.4. Data Analysis

CNR and SNR were calculated according to Szucs-Farkas et al. [18]

with

SNR = HU_artery_/noise_artery_

and

CNR = (HU_artery_ − HU_muscle_)/noise_air_

with noise being the standard deviation in the corresponding measurement.

The following statistical analyses were performed using R (4.2.2). A *p*-value less than 0.05 was considered statistically significant. For the results, categorial variables were shown as counts, and continuous variables as mean and standard deviation. Cohen’s Kappa was used to assess interrater reliability. A Kappa statistic between 0.61 and 0.8 was rated as substantial agreement, and above 0.81 as almost perfect according to Landis and Koch. A Kappa of 0 meant poor, between 0 and 0.2 slight, 0.21–0.4 fair, and 0.41–0.6 moderate agreement [19]. Analysis of variance (ANOVA) and post hoc *t*-tests were used to analyze objective image parameters for significant differences between the different energy levels. Vessels were grouped according to their size (less than 5 mm, 5–9 mm, 10–14 mm, 15–20 mm, and above 20 mm) and subgroup analyses were performed. Additionally, we performed ANOVA to check for relevant differences in objective parameters after grouping the vessels. The results were adjusted with correction factors depending on the Mauchly test of sphericity. If relevant differences were found, a post hoc *t*-test for dependent samples was executed.

## 3. Results

### 3.1. Patient Data

Our study included 14 male (70%) and 6 (30%) female patients with an average age of 55 years, ranging from 27 to 86 years. Further population data are shown in Table 2.

Due to a preceding surgery, one patient underwent a resection of the gastroduodenal artery. 

**Figure 1:**
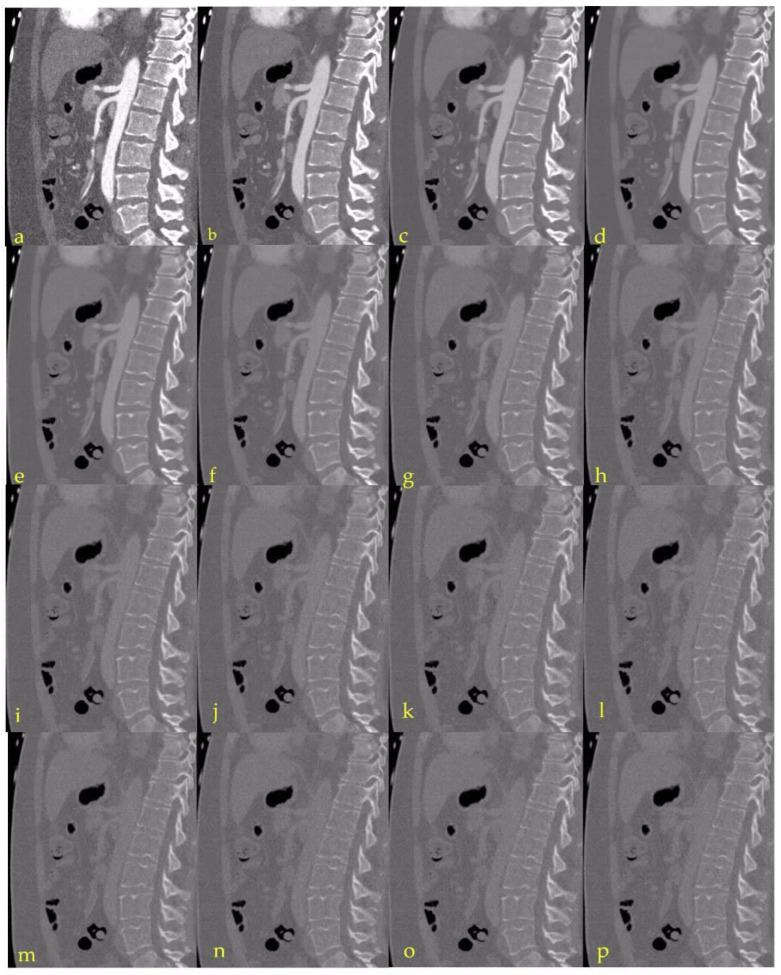
Sagittal images with focus on the abdominal aorta at different virtual monoenergetic levels to visualize the changes: (**a**) 40 keV; (**b**) 50 kV; (**c**) 60 keV; (**d**) 70 keV; (**e**) 80 keV; (**f**) 90 keV; (**g**) 100 keV; (**h**) 110 keV; (**i**) 120 keV; (**j**) 130 keV; (**k**) 140 keV; (**l**) 150 keV; (**m**) 160 kV; (**n**) 170 keV; (**o**) 180 keV; (**p**) 190 keV.

Figure 1 offers a general impression of the image changes with increasing virtual monoenergetic keV settings. 

### 3.2. Complete Dataset

#### 3.2.1. Attenuation

**Figure 2:**
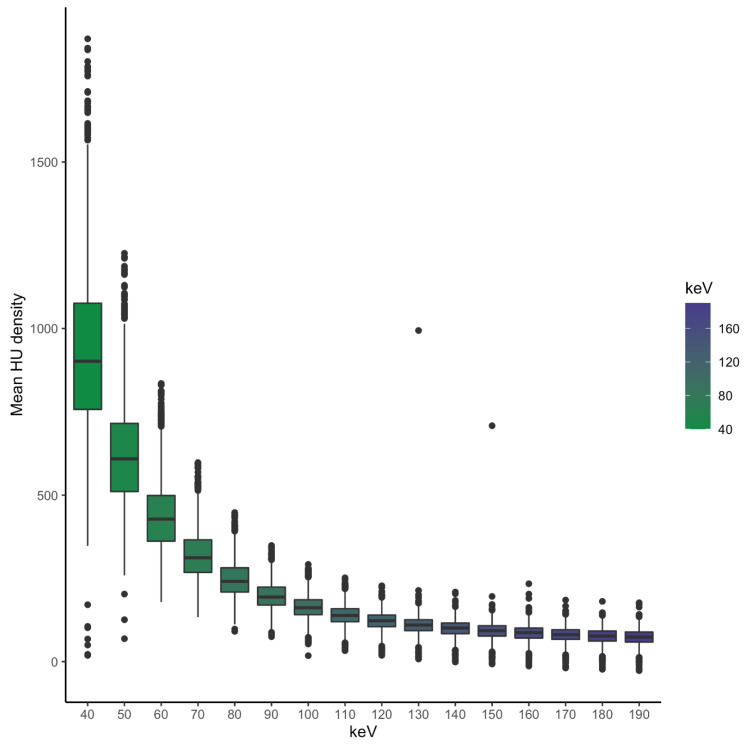
Decreasing mean vessel attenuation of all examined vessels with increasing virtual monoenergetic energy threshold levels.

The current study shows decreasing attenuation of the abdominal vessels with increasing energy threshold levels. Figure 2 shows the mean of all examined vessels with the corresponding energy level. 

Noise (standard deviation of the vessel attenuation) showed best values at the energy level of 80 keV (average of 27.5) and 70 keV (average of 28.4). Noise was highest at 40 keV (average of 119.1) and increased after a minimum at the energy levels of 70/80 keV to an average of 42 at the threshold of 190 keV.

#### 3.2.2. CNR

**Figure 3:**
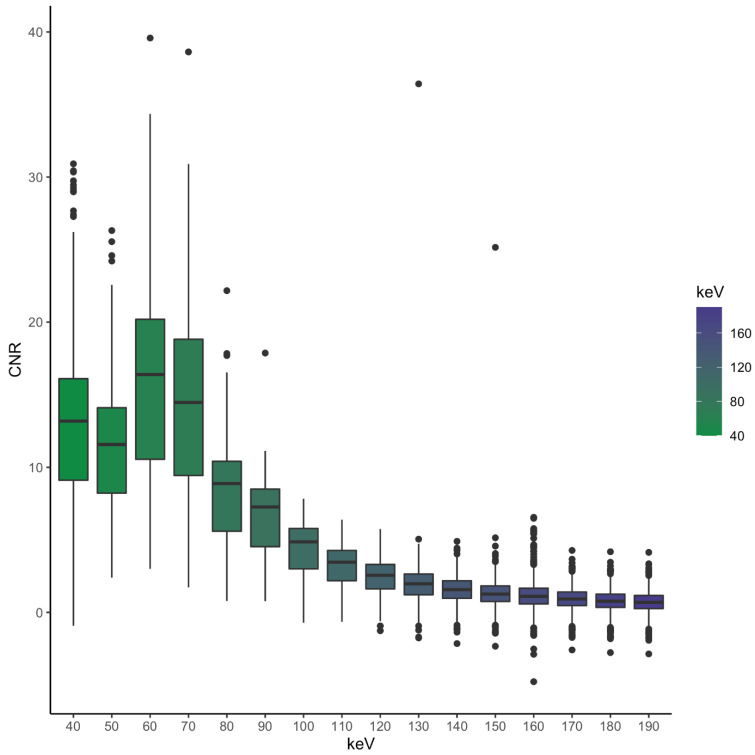
CNR of all examined vessels based on the VME threshold with a maximum at 60 keV.

The mean CNR in our study was highest at 60 keV (15.9) with a gradual decrease over higher energy levels (e.g., average at 80 keV: 8.27, at 90 keV: 6.71). CNR at 40 keV (13.1) was higher than at 50 keV (11.3) (*p* < 0.001), as shown in Figure 3.

#### 3.2.3. SNR

**Figure 4:**
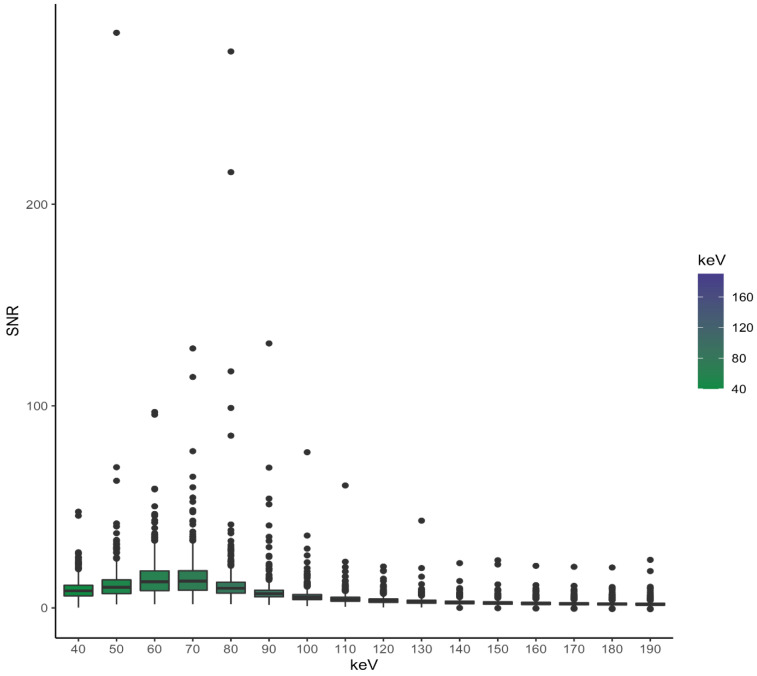
SNR of all arterial vessels grouped by the corresponding VME level; maxima can be seen at 60 keV and 70 keV.

SNR showed a flat curve with a maximum at 70 keV (average 15.0) and the second highest SNR at 60 keV (average 14.7), as shown in Figure 4. SNR showed no significant differences between the energy levels of 50 keV and 80 keV, and between 60 keV and 70 keV. All other compared energy thresholds were significantly different from each other regarding SNR, CNR and attenuation.

### 3.3. Grouping by Vessel Diameter

**Figure 5:**
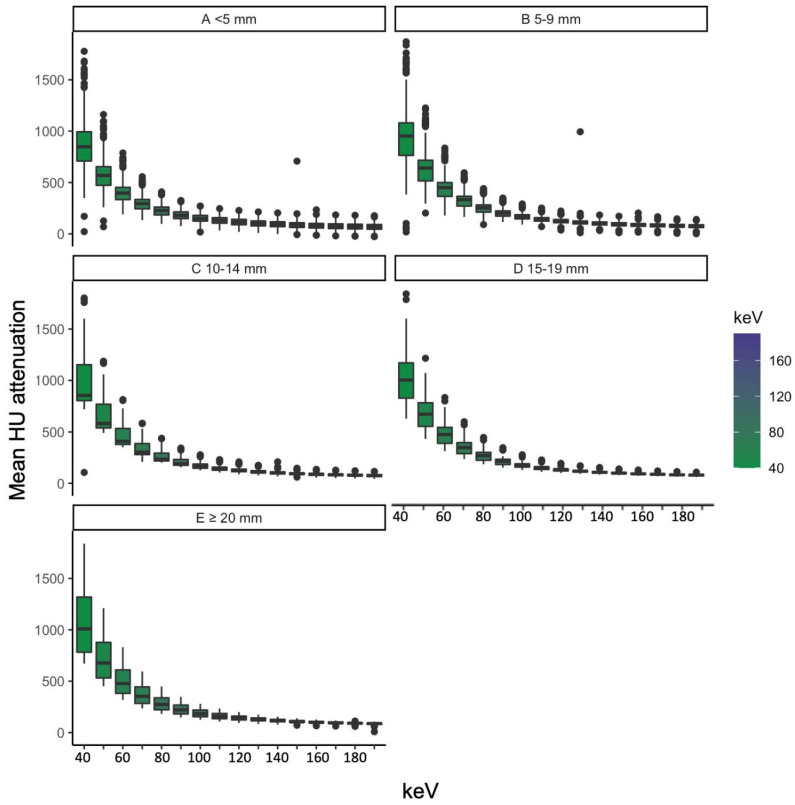
Attenuation grouped by vessel diameter ((**A**) for less than 5 mm, (**B**) for 5–9 mm, (**C**) for 19–14 mm, (**D**) for 15–19 mm and (**E**) for more than 20 mm) dependent on energy level.

**Figure 6:**
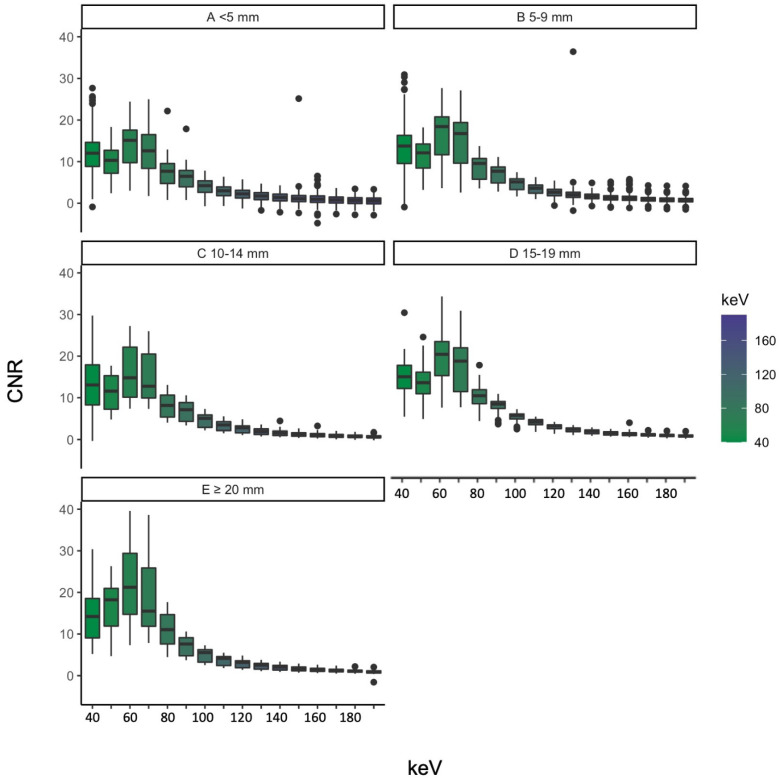
CNR grouped by vessel diameter ((**A**) for less than 5 mm, (**B**) for 5–9 mm, (**C**) for 19–14 mm, (**D**) for 15–19 mm and (**E**) for more than 20 mm) according to the used energy threshold.

**Figure 7:**
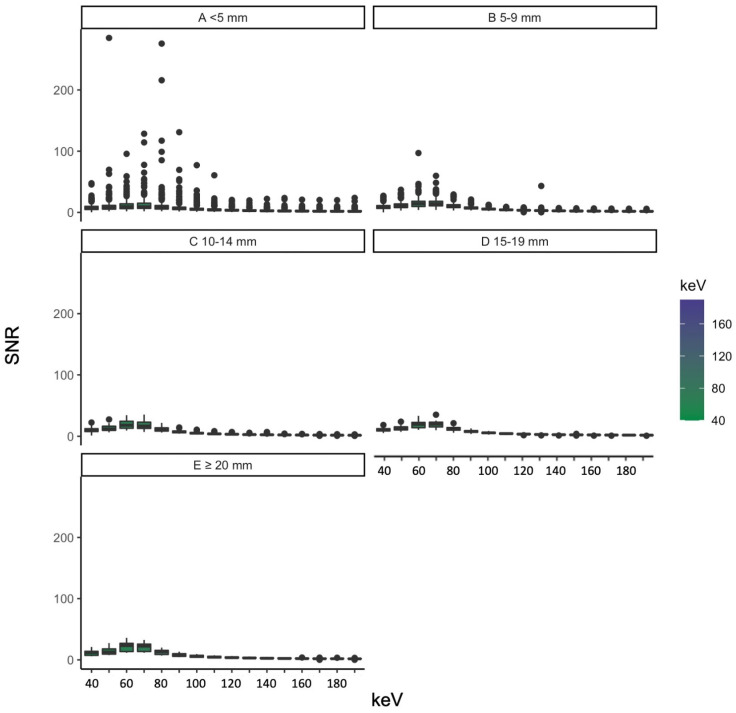
SNR grouped by vessel diameter ((**A**) for less than 5 mm, (**B**) for 5–9 mm, (**C**) for 19–14 mm, (**D**) for 15–19 mm and (**E**) for more than 20 mm) at different energy levels.

**Figure 8:**
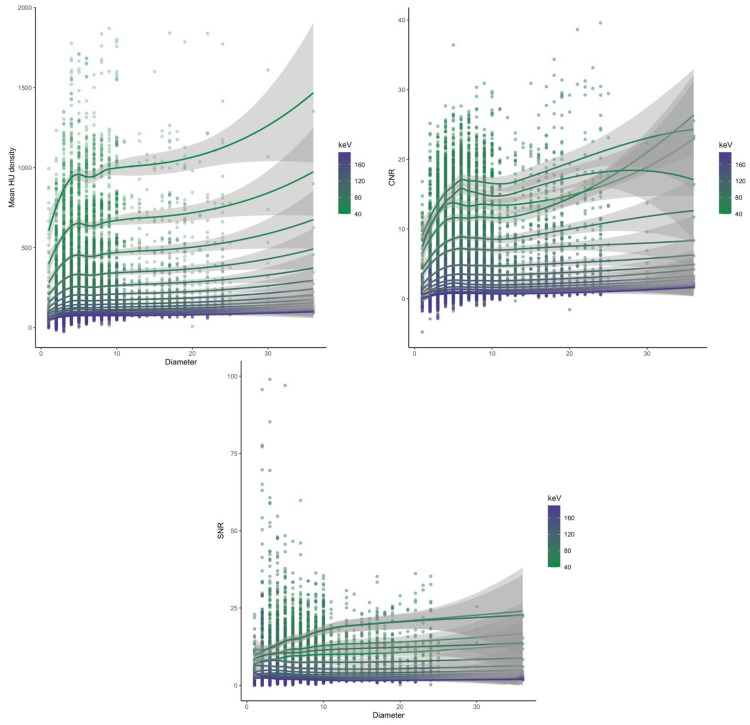
Scatterplots of the values of HU, CNR and SNR correlated with vessel diameter.

We grouped the objectively measured image parameters by vessel diameter and identified vessels smaller than 5 mm as the ones with the highest standard deviation of SNR. HU, CNR and SNR grouped by vessel diameter can be seen in Figure 5, Figure 6 and Figure 7. The scatterplots in Figure 8 visualize the spreading of the parameters with smaller vessel size.

#### 3.3.1. Attenuation

Overall attenuation was lower in smaller vessels which is visualized in Figure 3 (e.g., at 60 keV vessels smaller than 5 mm showed an average of 421 HU, and vessels between 5 and 9 mm an average of 451 HU, *p* < 0.001). 

Regarding attenuation, we found statistically relevant differences in all subgroups for all energy levels (see also Figure 5). 

#### 3.3.2. CNR

CNR showed a dip from 40 keV to 50 keV for vessels smaller than 19 mm and a gradual increase for vessels bigger than 20 mm between these two levels with a peak at 60 keV. This maximum is also visible for the smaller vessel diameters. 

Small vessels (<5 mm) showed no significant difference regarding CNR between the levels of 40 keV and 70 keV (*p* = 0.129); this was also seen for vessels between 10 and 14 mm (*p* = 0.190). Vessels between 15 and 20 mm showed no significant difference between the thresholds of 40 and 50 keV (*p* = 0.132) and 150/160 keV (*p* = 0.061). The biggest vessels (>20 mm) had significant differences at all energy thresholds besides 40/50 keV (*p* = 0.204), 40/70 keV (*p* = 0.187), 50/70 keV (*p* = 0.238) and 180/190 keV (*p* = 0.101). The CNR with the corresponding vessel size of the different energy levels can be seen in Figure 6. 

#### 3.3.3. SNR

The SNR curve was flatter for smaller vessel diameters than for vessels with diameters larger than 20 mm. 

For SNR, no statistically relevant differences regarding small vessels less than 5 mm could be seen at 40/90 keV (*p* = 0.672), 50/60 keV (*p* = 0.070), 50/80 keV (*p* = 0.546), 60/70 keV (*p* = 0.221), 60/80 keV (*p* = 0.525) and 70/80 keV (*p* = 0.158). The vessel diameter of 5–9 mm showed no significant differences between the levels of 60 and 70 keV (*p* = 0.946); the same could be seen for vessels between 10 and 14 mm (*p* = 0.497) and 15–20 mm (*p* = 0.213). The biggest vessel subgroup showed a *p*-value of 0.046 at this pairing. Vessels bigger than 20 mm showed no statistically relevant differences between the energy levels of 170/180 keV (*p* = 0.916), 170/190 keV (*p* = 0.156) and 180/190 keV (*p* = 0.052). SNR for different vessel sizes is shown in Figure 7. 

Noise optimum at 80 keV was statistically significant compared to 70 keV overall and in all vessel diameter subgroups. 

### 3.4. Subjective Parameters

Vessel contrast at 70 keV was rated best (with a 5 for each examination by both readers) with a gradual increase of the ratings up to this maximum and a decrease up to 190 keV. The levels 50–80 keV all showed at least one rating of 5. For 190 keV, all examinations were rated with the value of 1. 

Noise and image quality showed best values for both readers at 70 keV as well. The best rating of 5 for noise was only seen at the levels of 80 and 90 keV; overall image quality was given the best rating at the levels of 60–90 keV. Both show an increase up to 70 keV as with vessel contrast, and a decrease for energy levels above 70 keV.

The interobserver reliability with Cohen’s Kappa for subjective image noise showed fair agreement for both readers regarding image quality (0.237) and moderate interrater reliability for image noise (0.461) and vessel contrast (0.430). 

## 4. Discussion

The aim of our study was to evaluate VMI originating from spectral datasets of a photon-counting detector CT scanner regarding objective and subjective image quality, with a focus on abdominal vessels and an additional focus on their size. 

Our study showed significant differences regarding objective image parameters at almost all energy thresholds. The highest SNR was seen at 70 keV (with no significant difference to 60 keV). The best CNR was seen at 60 keV, which is in line with the findings of Martin et al. [20]. Noise was lowest at 80 keV, which is also in accordance with the findings of Sudarski et al. [1] for dual-source dual-energy CT. They also showed that SNR was highest at 70 keV regarding abdominal arteries even though there was no significant difference between monoenergetic and polyenergetic images in attenuation, contrast-to-noise ratio and noise overall. Albrecht et al. showed in a study with dual-energy imaging a superior CNR of monoenergetic images at 40–70 keV compared to the linearly blended images using Mono+ reconstruction [6]. 

In our study, attenuation increased with lower energy levels, resulting in an enhancement of vascular contrast, which can be explained by the approach to the k-edge of iodine and thus the absorption maximum [21,22]. This is also reflected by the increasing values of the Likert scale with lower keV. The smallest vessels showed the biggest standard deviation regarding SNR, which is most likely the result of some aberrant measurements which are shown in Figure 3. Due to smaller vessel sizes, the placed ROIs were naturally smaller as well, and therefore, bigger or smaller attenuations within the ROIs could lead to these aberrant values.

The standard window at 40 keV showed a blooming of the intravascular contrast medium which could lead to an overestimation of the vessel diameter and hide changes to the vessel wall, equivalently to Doerner et al. [23]. Despite offering the best vessel attenuation, image noise was elevated because the lower energy level is closer to the k-edge of iodine (33.2 keV) [21,22,24].

Buls et al. showed a decrease in contrast medium use and radiation at the energy level of 80 keV by using iterative reconstructions [25]. Therefore, optimizing the energy level by picking the most appropriate threshold in VMI instead of setting the tube voltage could result in the use of less contrast medium to obtain a feasible vessel contrast and therefore also result in less risk for the patient or vulnerable patients [21,25,26,27,28,29]. 

Additionally, higher keV levels showed better reduction in metal artifacts, for example for total hip replacements on a dual-layer CT scanner [30]. Even fewer metal artifacts were seen with special metal artifact reduction reconstruction algorithms [30,31,32,33]. Our study showed low intravascular contrast enhancement for energy levels above 100 keV with a slight decrease up to the maximum of 190 keV. This trend can be seen for all vessel subgroups, but these energy levels are important for metal artifact reduction [34]. Further studies should evaluate the best trade-off regarding metal artifact reduction and vessel contrast (e.g., in imaging of the runoff of both legs in peripheral arterial disease in patients with hip or knee replacements and consecutive metal artifacts). 

The interrater reliability showed only fair interobserver agreement for subjective image quality, but a clear optimum regarding image quality can be seen at 70 keV as both readers rated all examinations with a 5 at this energy level. This differs from other previous studies such as Graafen et al. [7] which showed best subjective parameters (e.g., objective image quality) at energy levels of 50 and 60 keV, maybe because our study not only included intravascular contrast but also the assessability of the vascular wall which suffers from increased noise at lower energy levels. Albrecht et al. also showed best subjective image quality results at 70 keV [35].

We also did not reconstruct images with different strengths of iterative reconstruction, which showed an improvement in CNR through noise reduction in the research of Sartoretti et al. [36]. Regarding subjective noise and vessel contrast, a peak can be seen at 70 keV along with moderate interobserver reliability. This differs from objective noise measurement (which was statistically best at 80 keV) but might correlate with the small absolute difference in noise between these two energy levels. 

Our study has some limitations, mostly due to its retrospective design and single-center character, but also to the number of patients included. Further studies with larger sample sizes are needed to confirm the results of our study. In addition, only bigger vessels (for example the aorta) should be examined to get more reliable and stable results as smaller vessels showed a few very high values (especially in SNR) which can be a result of the above-discussed smaller ROIs. In addition, different levels of iterative reconstruction should be considered for further research.

## 5. Conclusions

Our study suggests that photon-counting VMI is feasible to optimize the visualization of abdominal arteries. According to our data, VMI shows the best results for the combination of CNR, SNR, and vessel attenuation for VME images at an energy level of 60–70 keV.

## Figures and Tables

**Table 1:** Five-point Likert Scale for judging image quality, image noise and vessel contrast.

	Image Quality	Image Noise	Vessel Contrast
1	Very poor	Major noise	Not diagnostic
2	Poor	More than average noise	Poor
3	Acceptable	Average noise	Acceptable
4	Good	Minor noise	Good
5	Optimal	None	Optimal

**Table 2:** Population data including age, calculated patient diameter which correlates directly with body mass index [16], computed tomography dose index (CTDI vol), dose length product (DLP) and effective dose calculated as described in Section 2.1.

	Overall	Female	Male
Age (years)	55.15 ± 16	56.57 ± 12.18	54.38 ± 18.14
Diameter (cm)	30.68 ± 4.57	28.39 ± 4.12	31.91 ± 4.46
CTDI vol (mGy)	7.90 ± 3.92	5.14 ± 2.57	9.03 ± 3.85
DLP (mGy * cm)	330.57 ± 198.48	226.49 ± 155.8	374.36 ± 198.75
Effective Dose (mSv)	4.92 ± 2.97	3.33 ± 2.36	5.58 ± 2.96

## Data Availability

The data presented in this study is available on request from the corresponding author. The data is not publicly available due to ethical restrictions.
